# Transcriptome and DNA Methylation Analyses Provide Insight into Environmental Adaptation in Northern and Southern Populations of *Eriocheir sinensis*

**DOI:** 10.3390/ani16081164

**Published:** 2026-04-10

**Authors:** Junlei Ma, Mingxia Sun, Yidong Liang, Ting Zhang, Yulin Bai, Dongpo Xu

**Affiliations:** 1 Wuxi Fisheries College, Nanjing Agricultural University, Wuxi 214082, China; majunlei0304@gmail.com (J.M.);; 2 Key Laboratory of Freshwater Fisheries and Germplasm Resources Utilization, Ministry of Agriculture and Rural Affairs, Freshwater Fisheries Research Center, Chinese Academy of Fishery Sciences, Wuxi 214082, China; 3 National Demonstration Center for Experimental Fisheries Science Education, Shanghai Ocean University, Shanghai 201306, China

**Keywords:** Chinese mitten crab, DNA methylation, transcriptome, environmental adaptation, epigenetic regulation

## Abstract

The Chinese mitten crab (*Eriocheir sinensis*) is widely distributed along eastern China, where northern and southern populations may have evolved different environmental adaptation mechanisms. DNA methylation, as an important epigenetic modification, plays a key regulatory role in environmental adaptation. In this study, muscle and hepatopancreas tissues from *E. sinensis* populations of the Liaohe River (LH) and Beilunhe River (BLH) were subjected to integrated RNA-seq and whole-genome bisulfite sequencing (WGBS) analyses, generating 12 mRNA libraries and 12 DNA methylation libraries. RNA-seq analysis identified 622 and 783 differentially expressed genes (DEGs) in muscle and the hepatopancreas, respectively, in the LH group compared with the BLH group, with downregulated genes accounting for a larger proportion. WGBS analysis showed that genome-wide DNA methylation in *E. sinensis* was predominantly in the CG context, with the highest methylation levels observed in gene body regions. A total of 972 and 991 differentially methylated regions (DMRs) were identified in muscle and the hepatopancreas, respectively. Integrated analysis further identified 10 differentially methylated and expressed genes (DMEGs) in muscle and 26 DMEGs in the hepatopancreas. Notably, no single fixed pattern was observed between methylation changes and gene expression changes in either tissue. These findings suggest that DNA methylation may participate in environmental adaptation in northern and southern populations of *E. sinensis* by modulating gene expression. Our results highlight the important role of epigenetic regulation in the evolutionary adaptation of crustaceans to different environments and provide a theoretical basis for the development of environment-associated molecular markers and the evaluation of adaptive germplasm resources.

## Simple Summary

Chinese mitten crabs (*Eriocheir sinensis*) from northern and southern China inhabit distinct geographic regions characterized by different environmental conditions. In this study, we compared the transcriptome and DNA methylation profiles of muscle and hepatopancreas tissues between these northern and southern populations. We identified significant population-level differences in both tissues and found several genes where DNA methylation changes were associated with altered expression. These results suggest that epigenetic regulation may contribute to the environmental adaptation of *E. sinensis* across different latitudes. Our findings provide new insights into the molecular basis of population divergence in this species and identify potential candidate genes for future research on crustacean adaptation to complex environments.

## 1. Introduction

Complex environmental conditions, particularly temperature, are critical factors influencing the growth, development, reproduction, and distribution of ectothermic aquatic organisms, significantly impacting their physiological adaptability [1]. Ectothermic invertebrates are particularly susceptible to factors such as climate change and habitat temperature [2,3]. Environmental fluctuations disrupt cellular activities, exerting substantial effects on the survival and growth of aquatic animals [4]. Eurythermal aquatic organisms distributed across broad latitudes possess a wide environmental tolerance range and exhibit distinct population-level adaptive mechanisms that may be closely related to their specific local thermal environments [5].

Therefore, populations inhabiting different latitudes may possess heritable phenotypic differentiation or transcriptional regulatory mechanisms to respond to selective pressures. Latitude influences growth rates, basal metabolic rates, and reproductive investment in aquatic animals [6,7]. Studies reveal that *Cerastoderma edule* living at low latitudes primarily redirect energy toward gonadal development, spawning nearly year-round, resulting in slow growth and smaller body size [8]. High-latitude populations of the marine herbivorous butterfish (*Odax pullus*) and the carnivorous banded wrasse (*Notolabrus fucicola*) exhibit slower growth rates but ultimately reach larger body sizes than their low-latitude counterparts [9]. Muscle tissue constitutes the primary edible portion of aquatic animals, holding significant economic value. The muscle tissue of northern and white shrimp (*Litopenaeus vannamei*) efficiently generates and supplies energy for heat shock protein synthesis under elevated temperatures [10]. The hepatopancreas, serving as the core metabolic organ and energy storage center in crustaceans, plays a crucial regulatory role during thermal stress.

The Chinese mitten crab (*Eriocheir sinensis*) is one of the most economically important freshwater aquaculture crustaceans in China, with an annual production consistently exceeding 800,000 tons and generating immense commercial value. Characterized by its eurythermal and euryhaline traits, it is also an ideal model organism for studying the genetic mechanisms underlying environmental adaptation. In China, *E. sinensis* populations are divided into northern and southern groups based on latitude, including northern crabs, represented by the Liaohe River, Yellow River, Yangtze River, and Oujiang River, and southern crabs, represented by the Zhujiang River, Beilunhe River, and Nanliu River [11]. Among these, winter water temperatures in the Liaohe River can drop to 0 °C [12], while winter temperatures in the Beilunhe River remain above 14 °C [13]. Current research primarily employs basic genetics and morphology to provide limited insights into the population genetic structure and environmental adaptability of river crabs across different regions [11,14,15]. These approaches cannot fully explain the differences in environmental adaptability and morphological behavioral traits between northern and southern populations. Further exploration and analysis through gene expression and epigenetic regulation are necessary.

Epigenetically mediated phenotypic plasticity serves as a primary mechanism for species responding to changing environments and rapidly shifting climates [16]. Research has revealed that DNA methylation, as a form of epigenetic modification, plays a crucial role in species environmental adaptation. For instance, Platt et al. [17] discovered in natural populations of *Arabidopsis thaliana* that DNA methylation is closely associated with enhanced local environmental adaptation. Gao et al. [18] demonstrated that DNA methylation plays a crucial role in hypoxia regulation in the large yellow croaker (*Larimichthys crocea*). Gao et al. [19] revealed that DNA methylation participates in the molecular mechanism of low salinity adaptation in the swimming crab (*Portunus trituberculatus*) by regulating gene expression. Huang et al. [20] demonstrated that genetically improved farmed tilapia (GIFT, *Oreochromis niloticus*) adapts to high-salinity stress through DNA methylation by regulating vasoconstriction and energy metabolism. However, the role of epigenetic modifications, represented by DNA methylation, in environmental adaptation within natural populations of *E. sinensis* remains largely unexplored.

Therefore, to further elucidate the epigenetic mechanisms underlying adaptive differences between northern and southern populations of *E. sinensis*, this study employed whole-genome bisulfite sequencing (WGBS) and RNA-seq to analyze the epigenetic mechanisms of *E. sinensis* across different latitudes and environments. Concurrently, the findings will provide essential genetic research foundations for genetic breeding and germplasm innovation in *E. sinensis*.

## 2. Materials and Methods

### 2.1. Animals and Tissue Collection

**Figure 1:**
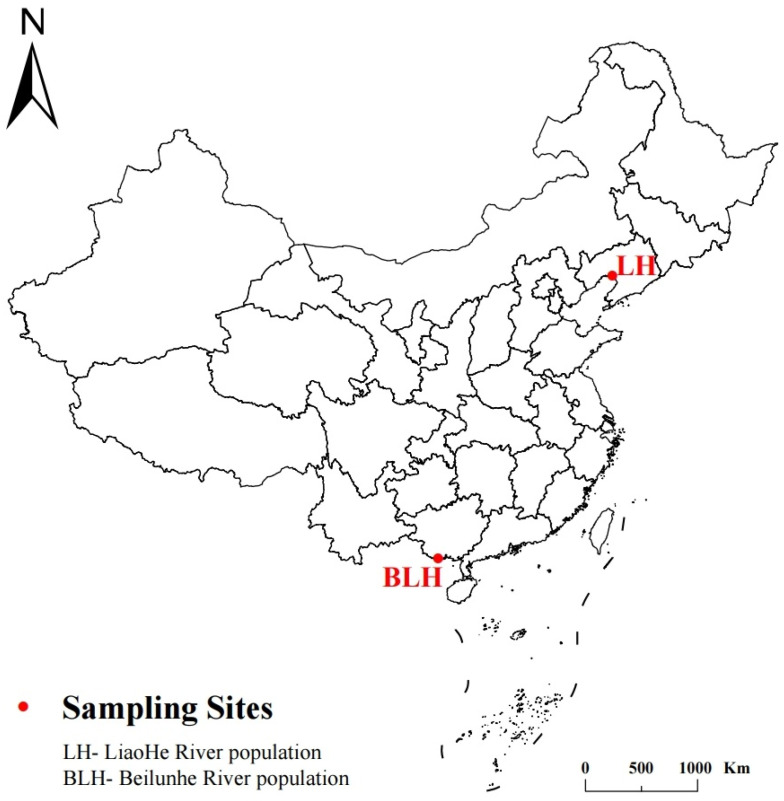
Sampling sites of the BLH and LH *Eriocheir sinensis* populations in China.

In October 2023, 6 wild-caught crab individuals from the Liaohe River population (LH) and 6 wild-caught crabs from the Beilunhe River population (BLH) were randomly selected from their natural habitats. The precise geographic locations of the two sampling sites along the eastern coast of China are illustrated in Figure 1. For the general environmental context, regional background records for October indicated that the LH region had an average water temperature of approximately 12 °C and a salinity of 0.5 ppt, whereas the BLH region had an average water temperature of approximately 26 °C and a salinity of 1.2 ppt. To evaluate and control for potential biological variability, key biological factors, including sex, body length, body width, body thickness, and body weight, were carefully measured prior to tissue collection. Both the LH and BLH groups consisted of an equal sex ratio (3 females and 3 males per population). Specifically, the biological parameters for the BLH population were: length: 45.96 ± 2.48 cm, width: 49.29 ± 1.97 cm, thickness: 22.53 ± 1.58 cm, and weight: 51.47 ± 7.54 g for females; length: 43.75 ± 1.34 cm, width: 46.22 ± 1.89 cm, thickness: 22.38 ± 0.71 cm, and weight: 53.18 ± 3.43 g for males. For the LH population, the parameters were: length: 44.04 ± 2.40 cm, width: 47.61 ± 2.45 cm, thickness: 23.81 ± 0.95 cm, and weight: 51.05 ± 7.57 g for females; length: 43.07 ± 1.90 cm, width: 47.06 ± 2.01 cm, thickness: 22.42 ± 1.43 cm, and weight: 50.95 ± 6.60 g for males. The detailed individual biometric parameters of all 12 selected crabs are summarized in Table 1.

After the crabs were anesthetized in an ice-water bath, hepatopancreas and muscle samples were collected and immediately frozen in liquid nitrogen for subsequent RNA or DNA extraction.

### 2.2. mRNA Library Construction and Sequencing

Total RNA was extracted from hepatopancreas and muscle tissues (*n* = 6 per group) from wild-caught *E. sinensis* of the LH and BLH populations using TRIzol Reagent (Invitrogen, Carlsbad, CA, USA) according to the manufacturer’s standard protocol. Briefly, tissues were homogenized in the reagent, followed by phase separation with chloroform, isopropanol precipitation, and a 75% ethanol wash. Then, equal amounts of extracted RNA from two individuals within the same group were mixed for sequencing, with 3 biological replicates per group. Taking the hepatopancreas as an example, 6 RNA-seq libraries were prepared (LH_L_1, LH_L_2, LH_L_3, BLH_L_1, BLH_L_2, and BLH_L_3). Muscle tissues were processed similarly to generate the corresponding libraries. The quality of total RNA was evaluated using an Agilent 5400 system (Agilent, Santa Clara, CA, USA) and a NanoDrop Lite spectrophotometer (NanoDrop Technologies, Wilmington, DE, USA).

For high-quality RNA, mRNA sequencing libraries were prepared with the RNA Library Prep Kit (Illumina, San Diego, CA, USA) for Illumina following the manufacturer’s guidelines. Initially, ribosomal RNA was eliminated, and the resulting rRNA-free residue was purified via ethanol precipitation. The purified RNA was then fragmented into short fragments, which served as templates for first-strand cDNA synthesis using reverse transcriptase. Subsequently, second-strand cDNA was synthesized to generate double-stranded cDNA. After end repair, A-tailing, and adaptor ligation, the ligation products were subjected to PCR amplification using adaptor-specific primers to enrich the cDNA fragments carrying sequencing adaptors at both ends. The amplified products were then purified and size-selected to construct the final cDNA libraries. After library quality assessment and quantification, qualified libraries were sequenced on an Illumina platform (Illumina, San Diego, CA, USA) using the paired-end 150 bp (PE150) strategy, according to the effective library concentration and the required data output.

### 2.3. Bioinformatics Processing and Transcriptome Data Analysis

Raw sequencing reads were processed to remove adapter sequences, reads with more than 5% of bases undetermined, and low-quality reads, generating high-quality clean data. Quality metrics, including Q20 and Q30, were calculated to ensure sequencing reliability. The HISAT2 software (v.2.2.1) was then applied to align the valid clean reads to the *Eriocheir sinensis* reference genome for subsequent analysis. Using StringTie (v.2.1.4), transcriptomes were first assembled individually for each sample, and the “-merge” feature was utilized to combine them. Gffcompare (v.0.12.1) was employed to compare and annotate the newly generated transcriptomes against known reference annotations.

The transcriptional expression levels of genes were quantified and normalized using the Fragments Per Kilobase of transcript per Million mapped reads (FPKM) metric. Differential expression analysis between the LH and BLH groups was performed with the edgeR package in R (v3.12.1). Genes meeting the stringent criteria of an adjusted *p*-value < 0.05, |log2(FoldChange)| > 1, and an FPKM > 10 were defined as significantly differentially expressed genes (DEGs). Finally, to determine the biological functions and signaling pathways associated with these DEGs, Gene Ontology (GO) and Kyoto Encyclopedia of Genes and Genomes (KEGG) functional enrichment analyses were conducted, with a significance threshold set at a *p*-value < 0.05.

### 2.4. DNA Methylation Library Preparation and Sequencing

Whole-genome bisulfite sequencing (WGBS) libraries were constructed using the same crab individuals as those used for RNA-seq. Genomic DNA was extracted from hepatopancreas and muscle tissues of wild-caught *E. sinensis* from the LH and BLH populations (*n* = 6 per group) using the DNeasy Tissue Kit (Qiagen, Hilden, Germany). DNA integrity, concentration, and purity were assessed via agarose gel electrophoresis, the Qubit^®^ DNA Assay Kit in a Qubit^®^ 2.0 Fluorometer (Life Technologies, Carlsbad, CA, USA), and a NanoDrop Lite spectrophotometer (NanoDrop Technologies, Wilmington, DE, USA), respectively. To maintain consistency with the RNA-seq design, equal amounts of genomic DNA from two individuals within the same group were pooled for library construction, with one female and one male combined in each pool, resulting in three biological replicates per group. Taking the hepatopancreas as an example, six WGBS libraries were prepared: LH_L_1, LH_L_2, LH_L_3, BLH_L_1, BLH_L_2, and BLH_L_3. Muscle tissues were processed in the same way to generate the corresponding libraries. A mixture of 100 ng genomic DNA and 0.5 ng lambda DNA was fragmented using a Covaris S220 (Covaris, Woburn, MA, USA) column. The fragmented DNA then underwent end repair, 3′-adenylation, and ligation of methylated adapters, followed by purification of the adaptor-ligated DNA fragments. Bisulfite conversion and PCR amplification were subsequently performed using the EZ DNA Methylation-Gold^TM^ Kit (Zymo Research, Irvine, CA, USA). Whole-genome methylation sequencing was conducted using the Illumina Nova platform (Illumina, San Diego, CA, USA) in 150 bp paired-end mode.

### 2.5. Methylation Calculation and Differentially Methylated Analysis

Using Bismark (v0.25.1), clean reads from whole-genome methylation sequencing were mapped to the *E. sinensis* genome. The mapped data were deduplicated using the Bismark Methylation Extractor to extract methylated cytosine sites. Conserved cytosine sites covered at least three times across all biological replicates were retained and imported into the R package methyKit (v.0.99.2) [21] for differential methylation analysis. Methylation levels were defined as mC reads/C reads. Differential methylation regions (DMRs) were identified using 1000 bp windows with 1000 bp steps. Regions showing significantly higher methylation levels in the LH group were defined as hyper-methylated (hyper) DMRs, while those with lower levels were defined as hypo-methylated (hypo) DMRs. Furthermore, based on the reference genome annotation, DMRs were categorized by their locations in upstream (promoter), exon, intron, gene_body, downstream, and intergenic regions. Significance thresholds were set at *q* < 0.01 and a differential percentage > 20%. Bedtools annotated genes and genomic elements within DMRs. Finally, GO and KEGG enrichment analyses were performed using the GOseq R package (v.1.50.0) [22] and KOBAS software (v.3.0) [23], respectively.

### 2.6. Conjoint Analysis of Methylome and Transcriptome Alterations

After obtaining DMGs, we used a custom script to compare them with DEGs derived from the transcriptome to identify differentially expressed differentially methylated genes (DMEGs) and explore their functional roles.

### 2.7. Experimental Validation

For qRT-PCR validation, 5 DEGs were selected from muscle and hepatopancreas tissues for experimental verification. Table S1 shows the primer sequences. The Mir-X ™ miRNA First-Strand Synthesis Reverse Transcription kit (TaKaRa, Dalian, China) was used to perform the reverse transcription of miRNA. The primers were synthesized by Sangon Biotech (Shanghai, China) Co., Ltd. The reaction system for miRNA qPCR amplification contained 2 × TB Green Advantage Premix (TaKaRa), 12.5 μL; miR-specific primer (10 μM), 0.5 μL; miRQ 3’ Primer (10 μM), 0.5 μL; cDNA, 2 μL; and H_2_O, 9.5 μL. Every sample was measured three times, with U6 serving as the reference gene. The CFX96 Real-Time PCR Detection System (Bio-Rad, Hercules, CA, USA) was used for the measurement, and the reaction procedure referred to the previous study [24].

The qRT-PCR methods for mRNA and lncRNA are briefly described as follows: The PrimeScript^TM^ RT reagent Kit (TaKaRa, Dalian, China) with gDNA Eraser and TB Green Premix Ex Taq^TM^‖ (TaKaRa, Dalian, China) kits were used for reverse transcription and RT-PCR, respectively. The reference gene was *β-actin*. The relative expression level was calculated using the 2^−ΔΔct^ method [25].

## 3. Results

### 3.1. Sequencing Data Overview of Northern and Southern Populations

RNA-seq and WGBS analyses were performed on the hepatopancreas and muscle tissues of *E. sinensis* collected from the LH and BLH populations. In total, 12 mRNA libraries and 12 WGBS methylation libraries were constructed, including LH_M1_1, LH_M1_2, LH_M1_3, LH_L_1, LH_L_2, LH_L_3, BLH_M_1, BLH_M_2, BLH_M_3, BLH_L_1, and BLH_L_2, BLH_L_3.

For RNA-seq, a total of 532,101,710 raw reads were generated, with an average of 44,341,809 raw reads per sample. After quality control, 524,483,678 clean reads were retained, with an average of 43,706,973 clean reads per sample, and were used for subsequent analyses. The Q20 values of all samples were higher than 96.74%, and the Q30 values were all above 91%. After mapping the clean reads to the reference genome of *E. sinensis*, the average mapping rate across samples was 76.03% (ranging from 58.37% to 82.99%), indicating good sequencing quality and mapping performance (Table S2).

For WGBS, a total of 2,006,616,194 raw reads were generated, with an average of 167,218,016 raw reads per sample. After quality control and deduplication, 458,222,531 clean reads were retained, with an average of 38,185,211 clean reads per sample. After mapping the clean reads to the reference genome of *E. sinensis*, the mapping rates of all samples ranged from 20.69% to 26.67% (Table S3).

### 3.2. Differential Expression Analysis of the RNA-Seq

**Figure 2:**
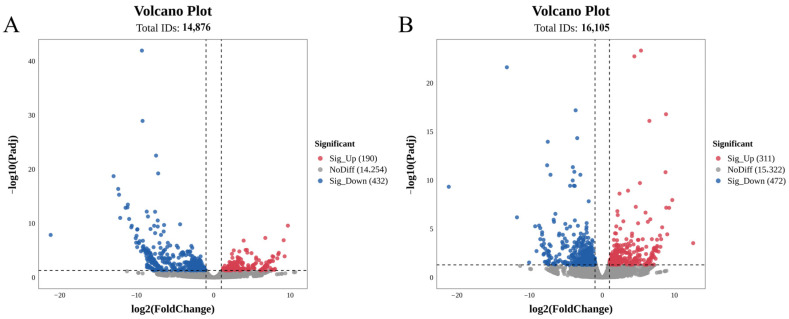
Differential expression analysis of mRNAs. (**A**) DE mRNA volcano plot in muscle of LH group compared with the BLH group; (**B**) DE mRNA volcano plot in hepatopancreas of LH group compared with the BLH group.

In the 12 mRNA libraries, genes with an adjusted *p*-value < 0.05, log_2_|FC| > 1, and FPKM > 10 were identified as DEGs. In muscle tissue, a total of 622 DEGs were detected in the LH group compared with the BLH group, among which 432 genes were downregulated, and 190 genes were upregulated, with downregulated genes being significantly more numerous than upregulated ones. In hepatopancreas tissue, a total of 783 DEGs were identified in the LH group relative to the BLH group, including 472 downregulated genes and 311 upregulated genes (Figure 2).

### 3.3. KEGG and GO Enrichment Analyses of DEGs

**Figure 3:**
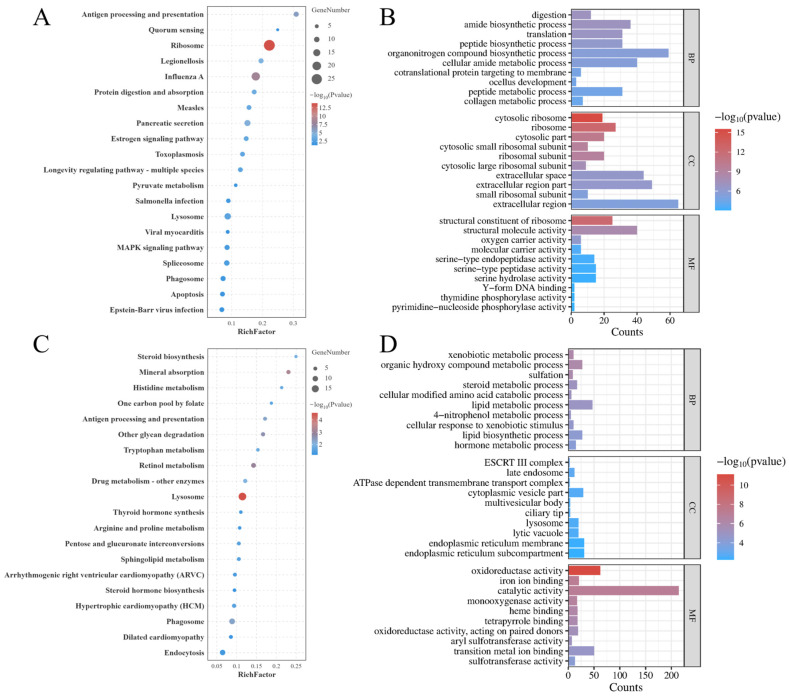
KEGG and GO enrichment analysis of DEGs of LH group compared with the BLH group. (**A**) Top 20 enriched KEGG pathways in muscle; (**B**) top 10 GO terms in three categories in muscle; (**C**) top 20 enriched KEGG pathways in hepatopancreas; (**D**) top 10 GO terms in three categories in hepatopancreas.

The KEGG enrichment analysis showed that, in muscle tissue, a total of 23 significantly enriched KEGG pathways were identified (Figure 3A), and the top 20 pathways are presented in the figure. The most significantly enriched pathway was the ribosome, which is a key pathway for protein synthesis. In addition, enrichment was also observed in immune-, metabolism-, and apoptosis-related pathways, including antigen processing and presentation, pancreatic secretion, apoptosis, protein digestion and absorption, and the MAPK signaling pathway. In hepatopancreas tissue, a total of 18 significantly enriched KEGG pathways were identified (Figure 3C), mainly involving immune- and metabolism-related pathways, such as lysosome, antigen processing and presentation, apoptosis, and phagosome, as well as metabolic pathways including amino acid metabolism and Steroid biosynthesis.

GO enrichment analysis revealed that the DEGs were mainly enriched in the three major categories: cellular components, molecular functions, and biological processes. The top 10 terms in each category are shown in Figure 3B,D. In muscle tissue, the DEGs were most significantly enriched in the cytosolic ribosome, followed by the ribosome. In hepatopancreas tissue, the DEGs were most significantly enriched in oxidoreductase activity, followed by iron ion binding and catalytic activity.

### 3.4. Differentially Methylated Region Analysis

**Figure 4:**
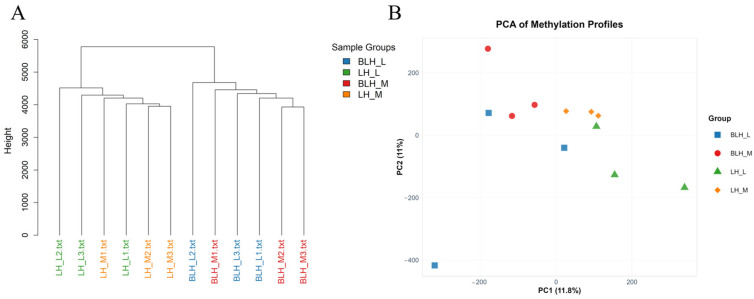
Correlations among the methylation level variations of CpG methylated regions in the 12 sequenced samples. (**A**) Hierarchical clustering analysis dividing samples into distinct population branches. (**B**) Principal Component Analysis (PCA) of methylation profiles showing clear geographic separation.

Clustering analysis of all methylation sequencing samples showed that the 12 samples were first separated into two distinct major branches, LH and BLH, according to geographic origin. Within each population branch, muscle and hepatopancreas tissues did not form clear tissue-specific subclusters (Figure 4A). PCA of inter-sample relationships revealed a clear separation along the PC1 axis based on geographic origin (Figure 4B), indicating that methylation differences between the northern and southern populations were substantially greater than those between hepatopancreas and muscle tissues.

**Figure 5:**
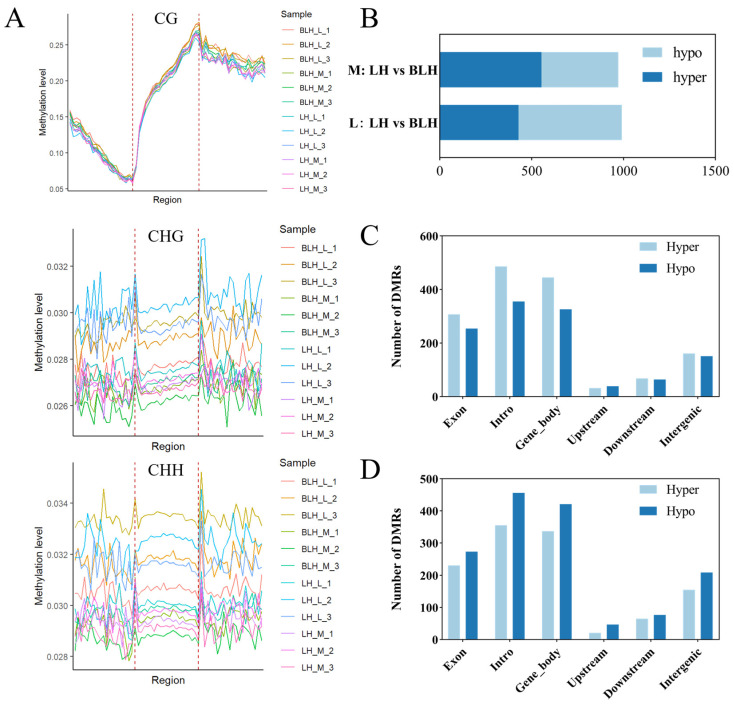
Differential methylation in the LH and BLH populations of *Eriocheir sinensis*. (**A**) Methylation levels for genes in different genetic regions at three mC content; (**B**) DMRs between different comparison groups; (**C**) distribution of differential methylation regions in muscles in different genetic regions under the CG context; (**D**) distribution of differential methylation regions in hepatopancreas in different genetic regions under the CG context.

Comparison of DNA methylation levels across different sequence contexts, including CG and the non-CG contexts CHG and CHH, where H represents adenine, cytosine, or thymine, showed that methylation in the CG context was predominant, whereas the average genome-wide methylation levels in the CHG and CHH contexts were both low (<5%) (Figure 5A). Therefore, subsequent analyses mainly focused on CG methylation. Comparison across different genomic regions further showed that methylation levels were highest in the gene body region, followed by the downstream region, and lowest in the promoter region.

DMR analysis showed that, compared with the BLH group, a total of 972 DMRs were identified in muscle tissue of the LH group, including 554 hype DMRs and 418 hypo DMRs. In hepatopancreas tissue, 991 DMRs were identified, including 429 hyper DMRs and 562 hypo DMRs (Figure 5B).

Further statistical analysis of the distribution of DMRs across different genomic functional regions showed that DMRs were widely distributed throughout the genome (Figure 5C,D). In muscle tissue, DMRs were mainly distributed in intron regions, gene body regions, and exon regions, with a relatively high proportion of hypo DMRs (Figure 5C). In hepatopancreas tissue, the distribution pattern of DMRs across genomic functional regions was generally similar to that in muscle tissue, but the proportion of hyper DMRs was higher in each region (Figure 5D).

### 3.5. KEGG and GO Enrichment Analyses of Annotated DMGs

**Figure 6:**
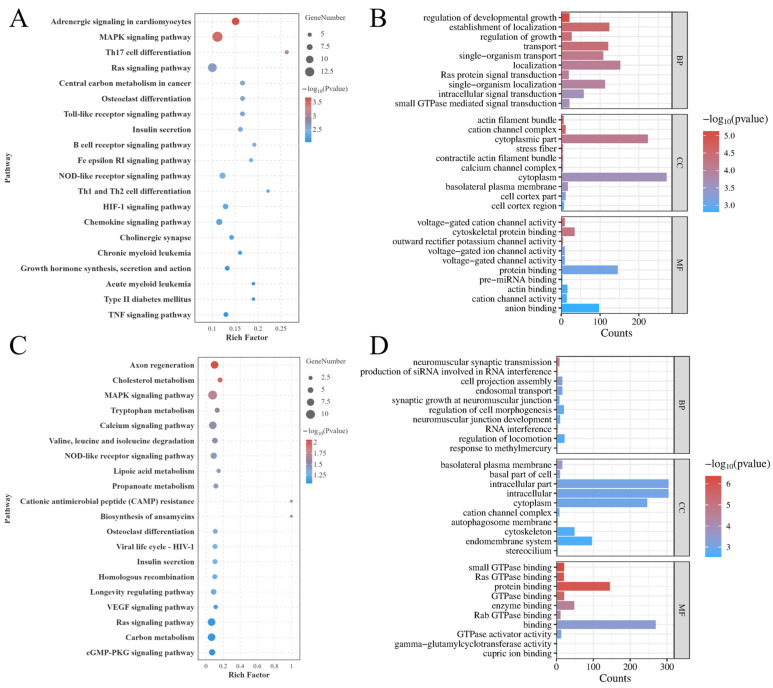
KEGG and GO enrichment analysis of DMGs of LH group compared with the BLH group. (**A**) Top 20 enriched KEGG pathways in muscle; (**B**) Top 10 GO terms in three categories in muscle; (**C**) top 20 enriched KEGG pathways in hepatopancreas; (**D**) top 10 GO terms in three categories in hepatopancreas.

KEGG and GO enrichment analyses were performed on the DMGs annotated from DMRs. KEGG enrichment analysis showed that, in muscle tissue, adrenergic signaling in cardiomyocytes was the most significantly enriched pathway. In addition, signal regulation pathways such as the MAPK signaling pathway and insulin signaling pathway were also significantly enriched (Figure 6A). In hepatopancreas tissue, the DMGs were mainly enriched in pathways related to signal transduction, metabolism, and regulation of cellular processes, such as axon regeneration, cholesterol metabolism and the MAPK signaling pathway (Figure 6C).

GO enrichment analysis showed that the DMGs were mainly enriched in the three major categories, cellular components, molecular functions, and biological processes, and the top 10 terms in each category are shown in Figure 6B,D. In muscle tissue, the DEGs were most significantly enriched in the regulation of developmental growth, followed by the establishment of localization and cytoplasmic parts (Figure 6B). In hepatopancreas tissue, the DMGs were mainly significantly enriched in MF categories such as small GTPase binding, ras GTPase binding, and protein binding (Figure 6D).

### 3.6. Integrated Analysis of Whole-Genome DNA Methylation and Transcriptome Data

**Figure 7:**
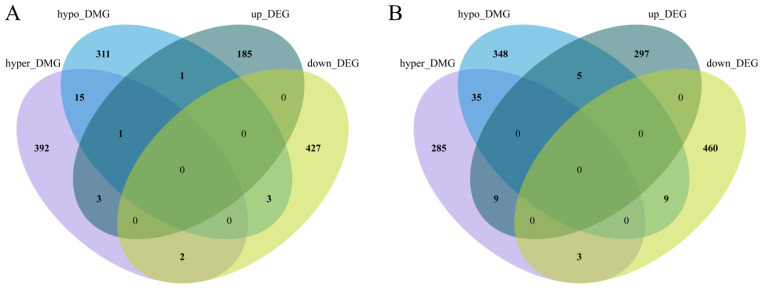
Combined genome-wide methylation and transcriptome analysis. (**A**) Muscle; (**B**) hepatopancreas.

By analyzing the overlap between DMGs and DEGs, we identified DEGs associated with DMRs. In muscle tissue between the LH and BLH groups, a total of 11 DMEGs were identified, including 2 hypo-methylated/upregulated DMEGs, 4 hyper-methylated/upregulated DMEGs, 3 hypo-methylated/downregulated DMEGs, and 2 hyper-methylated/downregulated DMEGs. In hepatopancreas tissue, a total of 26 DMEGs were identified, including 5 hypo-methylated/upregulated DMEGs, 9 hyper-methylated/upregulated DMEGs, 9 hypo-methylated/downregulated DMEGs, and 3 hyper-methylated/downregulated DMEGs (Figure 7). Among these DMEGs, *NESPRIN-1* and *SLC22A4* in muscle showed hypermethylation accompanied by downregulated expression, whereas *ATP1A* and CLCA2 were upregulated for gene expression. In the hepatopancreas, CAT and HAGO2 were downregulated in the LH group, whereas ABCC4, HECW2, and ANKIB1 were upregulated. Detailed information for all identified DMEGs, including their methylation and expression changes, is provided in Table S4. Among these DMEGs, *NESPRIN-1* and *SLC22A4* in muscle tissue showed hypermethylation accompanied by downregulated expression in the LH group. *ATP1A* also exhibited altered methylation status, together with downregulated expression, whereas *CLCA2* showed hypomethylation accompanied by downregulated expression in the LH group. In hepatopancreas tissue, *CAT* and *HAGO2* showed hypermethylation accompanied by downregulated expression in the LH group, whereas *ABCC4* showed hypermethylation accompanied by upregulated expression, and *HECW2*, *CLCN2* and *ANKIB1* showed hypomethylation accompanied by upregulated expression. Detailed information on the identified DMEGs, including their methylation and expression change patterns in each tissue, is provided in Table S4.

### 3.7. qRT-PCR Validation of DEGs

**Figure 8:**
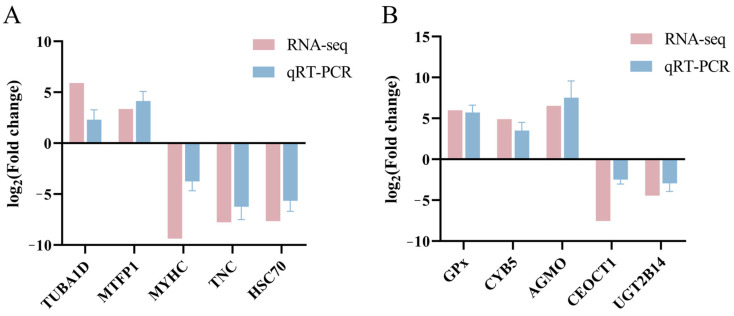
Confirmation by qRT-PCR of differential expression of 10 genes identified from RNA-Seq. (**A**) Comparison of log_2_ (fold change) values between RNA-Seq and qRT-PCR for five selected genes in muscle tissue. (**B**) Comparison of log_2_ (fold change) values between RNA-Seq and qRT-PCR for five selected genes in hepatopancreas tissue.

To validate the reliability of the transcriptome data, five DEGs from muscle tissue (*TUBA1D*, *MTFP1*, *MYHC*, *TNC*, and *HSC70*) and five DEGs from hepatopancreas tissue (*GPX*, *CYB5*, *AGMO*, *CEOCT1*, and *UGT2B14*) were selected for experimental verification. The sequencing results and the qRT-PCR verification results coincided closely for both the muscle (Figure 8A) and hepatopancreas (Figure 8B) tissues.

## 4. Discussion

In aquatic animals, successful adaptation to fluctuating environments is driven not only by genetic sequence variations but also by dynamic molecular regulatory mechanisms. Recent studies have shown that transcriptional regulation and epigenetic modifications can influence gene expression and phenotypic plasticity without altering the underlying gene sequence, thereby playing important roles in species adaptation to environmental conditions [26]. As one of the most classical and extensively studied epigenetic marks, DNA methylation can participate in organismal responses to abiotic stresses such as temperature, salinity, and acid–base conditions by regulating transcriptional activity of related genes and chromatin states [27,28,29,30]. The *E. sinensis* is widely distributed along the eastern regions of China from north to south, and the LH and BLH populations have long been exposed to distinct environmental backgrounds, which may have led to divergent adaptive strategies. Based on this, the present study employed RNA-seq and WGBS to investigate differences between northern and southern *E. sinensis* populations at the transcriptional and epigenetic levels.

Genome-wide cytosine methylation levels vary substantially among species. Previous studies have shown that the overall cytosine methylation level in arthropods is generally low, typically ranging from 0% to 1% [31,32], whereas methylation levels in fish, poultry, and mammals are usually around 3–10% [33,34,35,36]. In contrast, methylation levels in higher plants can reach as high as 30% [37]. In a previous study on sex-related methylation in *E. sinensis*, the methylation level was approximately 2.6–4.5% [38]. In the present study, the average methylation level in *E. sinensis* was about 4%, which is similar to that reported here, and higher than the 1.0–1.5% reported for swimming crab [19] and the 1.2% reported for Pacific white shrimp (*Litopenaeus vannamei*) [39]. These findings suggest that differences in methylation levels may be associated with species, populations, or environmental backgrounds.

From the perspective of sequence context, DNA methylation in animals is typically dominated by CpG (CG) sites [40], whereas non-CpG methylation (CHG and CHH) generally occurs at relatively low levels. In fish, CG methylation accounts for more than 90% of the three methylation types (CG, CHG, and CHH) [41]. Consistent with this pattern, methylation in *E. sinensis* in the present study occurred predominantly in the CG context, while methylation levels in the CHG and CHH contexts were low, which is in agreement with findings reported in Pacific white shrimp [39]. Meanwhile, our results showed that, at the level of genomic functional regions, differentially methylated regions were most abundant in the gene body (including exons and introns), followed by downstream regions, and least abundant in upstream regions. In invertebrates, gene body CG methylation is often considered to be associated with the maintenance of transcriptional stability, expression suppression, and alternative splicing, and may, therefore, be related to adaptation to different environments [42]. From a functional perspective, muscle is the primary tissue for energy expenditure and locomotor activity [43], whereas the hepatopancreas plays central roles in digestion, energy metabolism, and immune responses in crustaceans [44].

Transcriptomic data showed that, compared with the BLH population, the LH population had more downregulated than upregulated genes in both muscle and hepatopancreas tissues, indicating an overall trend of transcriptional suppression. This expression pattern may be associated with the regulation of physiological functions under the long-term environmental differentiation of northern and southern habitats [45]. In aquatic animals, integrated methylome and transcriptome studies have shown that contrasting environmental conditions, including salinity and other habitat-associated factors, can reshape gene expression and epigenetic states in a tissue-specific manner [39,46]. Therefore, the tissue-specific transcriptional differences observed here likely reflect distinct adaptive regulatory strategies shaped by contrasting environmental backgrounds between the LH and BLH populations.

To identify candidate genes associated with environmentally differentiated regulation between the two populations, we performed an integrated analysis of DMGs and DEGs and identified 11 and 26 DMEGs in muscle and hepatopancreas tissues, respectively. Previous studies have generally suggested that increased DNA methylation in promoters and regions near transcription start sites is often associated with transcriptional repression [47]. However, studies in species such as rice (*Oryza sativa*) and apple (*Malus domestica*) have also indicated that gene body methylation can be positively correlated with gene expression levels [48,49]. Consistent with these findings, we observed in the present study that methylation changes in different genes and different genomic regions led to distinct transcriptional responses, without a single uniform regulatory pattern. These results suggest that the relationship between DNA methylation and gene expression under different latitudinal environments is complex and may be jointly influenced by multiple factors, including the genomic location of methylation, methylation context type, magnitude of methylation change, and tissue/cell composition, as well as chromatin state.

In crustaceans, behaviors such as growth, locomotion, and feeding under different environments depend on the muscle tissue for power output, and both muscle contraction and neuromuscular transmission efficiency are highly sensitive to environmental changes [50,51]. In muscle tissue, the identified DMEGs were associated with muscle structure, ion transport, and metabolic regulatory pathways. *NESPRIN-1* is one of the core components of the LINC complex, linking the nucleus to the cytoskeleton and participating in myonuclear positioning and mechanotransduction [52]. Its hypermethylation and reduced expression in LH, therefore, suggest altered nucleo-cytoskeletal coupling and muscle structural remodeling under contrasting environmental backgrounds. Muscle contraction depends on action-potential-mediated excitation–contraction coupling [53,54]. *ATP1A* and *CLCA2* encode the Na^+^/K^+^-ATPase α subunit and a chloride channel accessory/regulatory factor, respectively [55,56]. Their upregulation in LH suggests strengthened ion transport and membrane homeostasis, which may help maintain muscle excitability. In addition, *SLC22A4* is an organic cation transporter capable of transporting small-molecule substrates such as carnitine and ergothioneine [57]. Its hypermethylation and downregulation in LH may indicate reduced transport of specific metabolic substrates.

The function of significantly DEGs in muscle tissue between the two populations further refines this interpretation. *TUBA1D*, an α-tubulin family gene, is closely associated with microtubule organization and cytoskeletal dynamics [58], and its upregulation is consistent with active structural remodeling in LH muscle. *MTFP1* is involved in mitochondrial dynamics and bioenergetic homeostasis [59], suggesting that mitochondrial regulation may also differ between populations. In contrast, *MYHC* and *TNC*, which encode core components of the contractile machinery and the Ca^2+^ sensor of the troponin complex, respectively [60,61], were downregulated in LH, implying reduced investment in contraction-related function. *HSC70* is an important molecular chaperone involved in protein folding and proteostasis [62], and its downregulation further suggests altered protein quality control in muscle. Taken together, both the DMEGs and qRT-PCR-validated genes indicate that muscle divergence between the two populations may mainly involve coordinated adjustment of cytoskeletal organization, ion homeostasis, mitochondrial dynamics, and contractile activity under contrasting environmental backgrounds.

The crustacean hepatopancreas plays central roles in digestion and nutrient metabolism, immune defense, and the maintenance of internal homeostasis and is, therefore, widely regarded as a key organ involved in environmental adaptation [10,63]. The identified DMEGs suggested that divergence between LH and BLH mainly involved antioxidant regulation, transmembrane transport, protein turnover, and post-transcriptional control in hepatopancreas. Catalase (CAT), a key antioxidant enzyme responsible for eliminating H_2_O_2_, is commonly used as an indicator of oxidative stress in studies of environmental adaptation in crustaceans [64]. In the present study, *CAT* in the LH population showed hypomethylation together with downregulated expression, suggesting that *E. sinensis* may adopt different antioxidant strategies under different environments. *ABCC4* is an ABC transporter involved in the efflux of organic anions and signaling molecules [65], whereas *CLCN2* is associated with chloride transport and ionic homeostasis. Their upregulation in LH suggests strengthened transmembrane transport and homeostatic regulation. *HECW2* and *ANKIB1* are both associated with ubiquitin-dependent protein turnover [66,67], indicating possible differences in protein quality control. In addition, regarding post-transcriptional regulation, *HAGO2* is a core component of miRNA-mediated gene silencing and post-transcriptional regulation [68]. and its downregulation suggests altered RNA-mediated regulatory activity in LH hepatopancreas.

The function of significant DEGs in the transcriptome results further refined this interpretation. GPX is a major glutathione-dependent antioxidant enzyme that reduces peroxides [69], and its upregulation indicates enhanced antioxidant defense. *CYB5* participates in fatty acid desaturation, sterol metabolism, and microsomal redox reactions [70], whereas AGMO is a key enzyme in ether lipid metabolism [71]; their increased expression suggests shifts in lipid-associated metabolism and redox regulation. By contrast, *CEOCT1*, an OCT1-like transporter, is functionally related to organic cation uptake [72], and *UGT2B14* belongs to the UDP-glucuronosyltransferase family, which catalyzes glucuronidation reactions important for detoxification [73]. Their downregulation suggests reduced substrate uptake and metabolic processing. Overall, both DMEGs and validated DEGs indicate that hepatopancreatic divergence between the two populations is primarily associated with coordinated remodeling of antioxidant capacity, transport processes, proteostasis, and metabolic detoxification under contrasting environmental backgrounds.

Furthermore, it should be noted that although this study highlights the potential role of DNA methylation in environmental adaptation, the observed differences in DNA methylation and gene expression between the LH and BLH populations may not be driven solely by environmentally associated regulation [74]. Long-term geographic separation between northern and southern populations may also have contributed to genetic background divergence [75]. Such genetic variation, including SNPs and structural variants, may influence methylation and gene expression patterns through regulatory effects, for example, meQTLs or eQTLs. Moreover, because the present study was based on a comparison of two natural populations rather than controlled-environment experiments, the current design does not allow the effects of temperature or other specific environmental variables to be disentangled from population-specific background differences. Future studies integrating whole-genome resequencing with epigenomic and transcriptomic data, ideally under common-garden and controlled-environment designs, will help clarify the relative contributions of genetic background and environmentally associated epigenetic variation in *E. sinensis*.

## 5. Conclusions

Overall, this study systematically investigated the environmental adaptation mechanisms of northern (LH) and southern (BLH) populations of *E. sinensis* at both the DNA methylation and transcriptional levels. Integrated analysis further identified DMEGs: those in muscle tissue were mainly involved in structural maintenance and ion homeostasis, whereas those in hepatopancreas tissue were primarily associated with antioxidant capacity, ion transport, and protein homeostasis. These findings provide an initial framework for understanding the complex regulatory network linking gene expression and DNA methylation during latitudinal environmental adaptation in northern and southern populations and offer an important basis for subsequent functional validation and further investigation of key candidate genes.

## Figures and Tables

**Table 1:** Biological parameters of the sampled *Eriocheir sinensis* individuals from the BLH and LH populations.

Sample ID	Sex	Length (cm)	Width (cm)	Thickness (cm)	Weight (g)
BLH-1	F	48.15	50.2	24.1	57.89
BLH-2	M	42.2	46.22	21.89	49.72
BLH-3	F	46.46	50.63	22.56	53.36
BLH-4	M	44.57	44.33	22.06	53.26
BLH-5	F	43.26	47.03	20.94	43.17
BLH-6	M	44.49	48.1	23.19	56.57
LH-1	M	42.86	46.33	22.34	51.96
LH-2	F	46.24	50.11	24.74	59.22
LH-3	M	45.06	49.33	23.89	56.99
LH-4	F	44.39	47.5	23.86	49.67
LH-5	M	41.28	45.51	21.04	43.91
LH-6	F	41.48	45.22	22.84	44.27

## Data Availability

The transcriptome sequencing data (mRNA libraries) have been deposited in the NCBI Sequence Read Archive (SRA) under BioProject accession number PRJNA1430100. The whole-genome bisulfite sequencing (WGBS) data have been deposited in the NCBI Sequence Read Archive (SRA) under BioProject accession number PRJNA1431406.

## Supplementary Materials

The following supporting information can be downloaded at https://www.mdpi.com/article/10.3390/ani16081164/s1: Table S1. Summary of data processing of mRNA libraries of Chinese mitten crab in LH and BLH groups. Table S2. Summary of data processing of DNA methylation libraries of Chinese mitten crab in LH and BLH groups. Table S3. Summary of DEMGs. Table S4. Summary of DEMGs.
