# Supplementary material for: Transcriptome and DNA Methylation Analyses Provide Insight into Environmental Adaptation in Northern and Southern Populations of Eriocheir sinensis
Source: Animals (Basel). 2026 Apr 10;16(8):1164. doi: 10.3390/ani16081164 (PMC13113693; doi:10.3390/ani16081164)
Supplement: Supplementary file 1 [file animals-16-01164-s001.zip › Table S1. Summary of data processing of mRNA libraries of Chinese mitten crab in LH and BLH groups.pdf]

Table S1. Summary of data processing of mRNA libraries of Chinese mitten crab in LH and BLH groups

Sample	Raw reads	Clean reads		Clean reads ratio (%)	Q20 (%)	Q30(%)	GC content%
	Read	Read	Base				
LH_M1	42048344	41322988	6067415828	98.27	96.74	91.0	51.01
LH_M2	54327572	53495214	7847991984	98.47	97.01	91.65	45.08
LH_M3	50209748	49494474	7189925530	98.58	97.22	92.20	44.57
LH_L1	45834480	45198676	6617522386	98.61	97.60	93.34	49.89
LH_L2	45004594	44423330	6462009582	98.71	97.68	93.45	51.48
LH_L3	45722366	45126676	6597203440	98.70	97.42	92.77	51.21
BLH_M1	46969868	46279998	6827337448	98.53	97.68	93.45	46.14
BLH_M2	39337710	38730484	5691891176	98.46	97.42	92.77	52.04
BLH_M3	41007120	40341340	5892771728	98.38	98.26	95.10	51.37
BLH_L1	40681958	40104008	5905157714	98.58	97.99	94.41	50.64
BLH_L2	40517296	39997346	5829535732	98.72	97.39	92.71	51.57
BLH_L3	40440654	39969144	5806006402	98.83	97.23	92.27	52.68
