# Supplementary material for: Transcriptome and DNA Methylation Analyses Provide Insight into Environmental Adaptation in Northern and Southern Populations of Eriocheir sinensis
Source: Animals (Basel). 2026 Apr 10;16(8):1164. doi: 10.3390/ani16081164 (PMC13113693; doi:10.3390/ani16081164)
Supplement: Supplementary file 1 [file animals-16-01164-s001.zip › Table S2. Summary of data processing of DNA methylation libraries of Chinese mitten crab in LH and BLH groups.pdf]

Table S2. Summary of data processing of DNA methylation libraries of Chinese mitten crab in LH and BLH groups

Sample	Raw data		Clean data		Clean data ratio (%)	Mapped reads	Unique Mapping Rate (%)	Genome-wide methylation (%)
	Reads	Base	Reads	Base				
LH_M1	164206177	24.63G	159879240	23.03G	93.50	39596792	24.77	3.92
LH_M2	165379124	24.81G	160747899	23.20G	93.55	39622606	24.65	3.92
LH_M3	167856027	25.18G	163228513	23.61G	93.75	40203860	24.63	3.76
LH_L1	163877146	24.58G	158949039	22.97G	93.45	39491431	24.85	3.94
LH_L2	155014315	23.25G	150718796	21.46G	92.30	36353044	24.12	4.17
LH_L3	164267236	24.64G	159942543	22.89G	92.90	38842631	24.29	4.02
BLH_M1	150078608	22.51G	145874899	21.14G	93.85	33941998	26.27	3.90
BLH_M2	167159997	25.07G	161508085	23.33G	93.05	37563109	23.26	3.81
BLH_M3	175950312	26.39G	170309317	24.54G	93.00	39642193	23.28	3.93
BLH_L1	170646189	25.60G	164580371	23.72G	92.65	37303562	22.67	4.06
BLH_L2	168949415	25.34G	163258340	23.22G	91.65	33779892	20.69	4.20
BLH_L3	193231648	28.98G	187401148	26.65G	91.95	41881413	22.35	4.34
