# Supplementary material for: Transcriptome and DNA Methylation Analyses Provide Insight into Environmental Adaptation in Northern and Southern Populations of Eriocheir sinensis
Source: Animals (Basel). 2026 Apr 10;16(8):1164. doi: 10.3390/ani16081164 (PMC13113693; doi:10.3390/ani16081164)
Supplement: Supplementary file 1 [file animals-16-01164-s001.zip › Table S3. Summary of DEMGs.pdf]

Table S3. Summary of DEMGs.

Common Group	Gene ID	Symbol	Hyper/Hypo	Region	Expression	Putative Function
LH_M vs. BLH_M	LOC126982254	*NESPRIN-1*	Hyper	Gene_body	down	Nuclear envelope spectrin repeat protein 1
	LOC126986769		Hypo	Gene_body/ Downstream	down	
	LOC126995008		Hypo	Gene_body	down	
	LOC126999269	*NFX1*	Hyper	Gene_body	up	NFX1-type zinc finger-containing protein 1-like
	LOC127000230	*CLCA2*	Hypo	Gene_body/ Downstream	up	putative calcium activated chloride channel regulator 2
	LOC127003085		Hyper	Gene_body	up	
	LOC127003200	*SLC22A4*	Hyper	Gene_body/ Downstream	down	organic cation transporter protein
	LOC127005145	*ATP1A*	Hyper/Hypo	Gene_body	up	Sodium/potassium-transporting ATPase subunit alpha
	LOC127005234		Hypo	Gene_body/ Downstream	up	
	LOC127008547	*NLRP10*	Hypo	Gene_body	down	NACHT, LRR and PYD domains-containing protein
LH_L vs. BLH_L	LOC126981298	*CLCN2*	Hypo	Gene_body	up	Chloride channel protein 2
	LOC126983631	*ANKIB1*	Hypo	Gene_body	up	Ankyrin repeat and IBR domain-containing protein 1
	LOC126984786	*MYO15*	Hypo	Gene_body	down	Unconventional myosin-15
	LOC126985153		Hypo	upstream	down	
	LOC126985306	*CFAP410*	Hypo	Gene_body/ Downstream	down	Cilia- and flagella-associated protein 410
	LOC126985859		Hyper	Gene_body	up	
	LOC126985867		Hyper	Gene_body	up	
	LOC126987851		Hypo	Gene_body	down	
	LOC126988022	*HAGO2*	Hyper	Gene_body	down	Protein argonaute-2
	LOC126995258		Hypo	Gene_body	up	
	LOC126995842	*CCDC53*	Hyper	Gene_body	down	Coiled-coil domain containing 53
	LOC126997334	*PXN*	Hyper	upstream	up	Paxillin
	LOC126997648		Hyper	Gene_body	up	
	LOC126998252		Hypo	Gene_body	down	
	LOC126998752	*DNAH3*	Hyper	Gene_body	up	dynein axonemal heavy chain 3
	LOC126999253		Hyper	Gene_body	down	
	LOC127001632	*CAT*	Hypo	Gene_body	down	Catalase
	LOC127002536	*HECW2*	Hyper	upstream	up	E3 ubiquitin-protein ligase

					HECW2
LOC127002834		Hyper	Downstream	up	
LOC127002867		Hypo	upstream	up	
LOC127002980		Hypo	Gene_body	up	
LOC127007428	*ABCC4*	Hyper	Gene_body	up	ATP-binding cassette sub-family C member 4
LOC127008335		Hypo	upstream	down	
LOC127009067	*CSTF1*	Hyper	Gene_body	up	cleavage stimulation factor subunit 1
LOC127009804		Hypo	Gene_body	down	
LOC127009806		Hypo	Gene_body	up	
