# Supplementary material for: Transcriptome and DNA Methylation Analyses Provide Insight into Environmental Adaptation in Northern and Southern Populations of Eriocheir sinensis
Source: Animals (Basel). 2026 Apr 10;16(8):1164. doi: 10.3390/ani16081164 (PMC13113693; doi:10.3390/ani16081164)
Supplement: Supplementary file 1 [file animals-16-01164-s001.zip › Table S4. Summary of DEMGs.pdf]

Table S4. Summary of DEMGs.

Common Group	Gene ID	Symbol	Hyper/Hypo	Region	log2FC (RNA-seq)	Expres sion	Putative Function
LH_M vs. BLH_M	LOC126982254	*NESPRIN-1*	Hyper	Gene_body	-1.32	down	Nuclear envelope spectrin repeat protein 1
	LOC126986769		Hypo	Gene_body/ Downstream	-7.65	down	
	LOC126995008		Hypo	Gene_body	-2.37	down	
	LOC126999269	*NFX1*	Hyper	Gene_body	2.35	up	NFX1-type zinc finger-containing protein 1-like
	LOC127000230	*CLCA2*	Hypo	Gene_body/ Downstream	2.19	up	putative calcium activated chloride channel regulator 2
	LOC127003085		Hyper	Gene_body	3.72	up	
	LOC127003200	*SLC22A4*	Hyper	Gene_body/ Downstream	-2.76	down	organic cation transporter protein
	LOC127005145	*ATP1A*	Hyper/Hypo	Gene_body	1.37	up	Sodium/potassium-transporting ATPase subunit alpha
	LOC127005234		Hypo	Gene_body/ Downstream	3.08	up	
	LOC127008547	*NLRP10*	Hypo	Gene_body	-2.70	down	NACHT, LRR and PYD domains-containing protein
LH_L vs. BLH_L	LOC126981298	*CLCN2*	Hypo	Gene_body	1.28	up	Chloride channel protein 2
	LOC126983631	*ANKIB1*	Hypo	Gene_body	2.26	up	Ankyrin repeat and IBR domain-containing protein 1

LOC126984786	*MYO15*	Hypo	Gene_body	-2.44	down	Unconventional myosin-15
LOC126985153		Hypo	upstream	-3.75	down	
LOC126985306	*CFAP410*	Hypo	Gene_body/ Downstream	-2.14	down	Cilia- and flagella-associated protein 410
LOC126985859		Hyper	Gene_body	1.20	up	
LOC126985867		Hyper	Gene_body	1.02	up	
LOC126987851		Hypo	Gene_body	-2.40	down	
LOC126988022	*HAGO2*	Hyper	Gene_body	-2.44	down	Protein argonaute-2
LOC126995258		Hypo	Gene_body	4.78	up	
LOC126995842	*CCDC53*	Hyper	Gene_body	-1.21	down	Coiled-coil domain containing 53
LOC126997334	*PXN*	Hyper	upstream	1.21	up	Paxillin
LOC126997648		Hyper	Gene_body	2.09	up	
LOC126998252		Hypo	Gene_body	-2.03	down	
LOC126998752	*DNAH3*	Hyper	Gene_body	3.31	up	dynein axonemal heavy chain 3
LOC126999253		Hyper	Gene_body	-2.42	down	
LOC127001632	*CAT*	Hypo	Gene_body	-2.07	down	Catalase
LOC127002536	*HECW2*	Hyper	upstream	2.23	up	E3 ubiquitin-protein ligase HECW2
LOC127002834		Hyper	Downstream	4.41	up	
LOC127002867		Hypo	upstream	1.91	up	
LOC127002980		Hypo	Gene_body	1.53	up	
LOC127007428	*ABCC4*	Hyper	Gene_body	1.56	up	ATP-binding cassette sub-family C member 4
LOC127008335		Hypo	upstream	-1.73	down	

LOC127009067	*CSTF1*	Hyper	Gene_body	4.08	up	cleavage stimulation factor subunit 1
LOC127009804		Hypo	Gene_body	-1.42	down	
LOC127009806		Hypo	Gene_body	-1.59	up	
